# Supplementary material for: Hetero-type dual photoanodes for unbiased solar water splitting with extended light harvesting
Source: Nat Commun. 2016 Dec 14;7:13380. doi: 10.1038/ncomms13380 (PMC5477502; doi:10.1038/ncomms13380)
Supplement: Supplementary Information — Supplementary Figures 1-19, Supplementary Tables 1-2, Supplementary Discussion and Supplementary References. [file ncomms13380-s1.pdf]

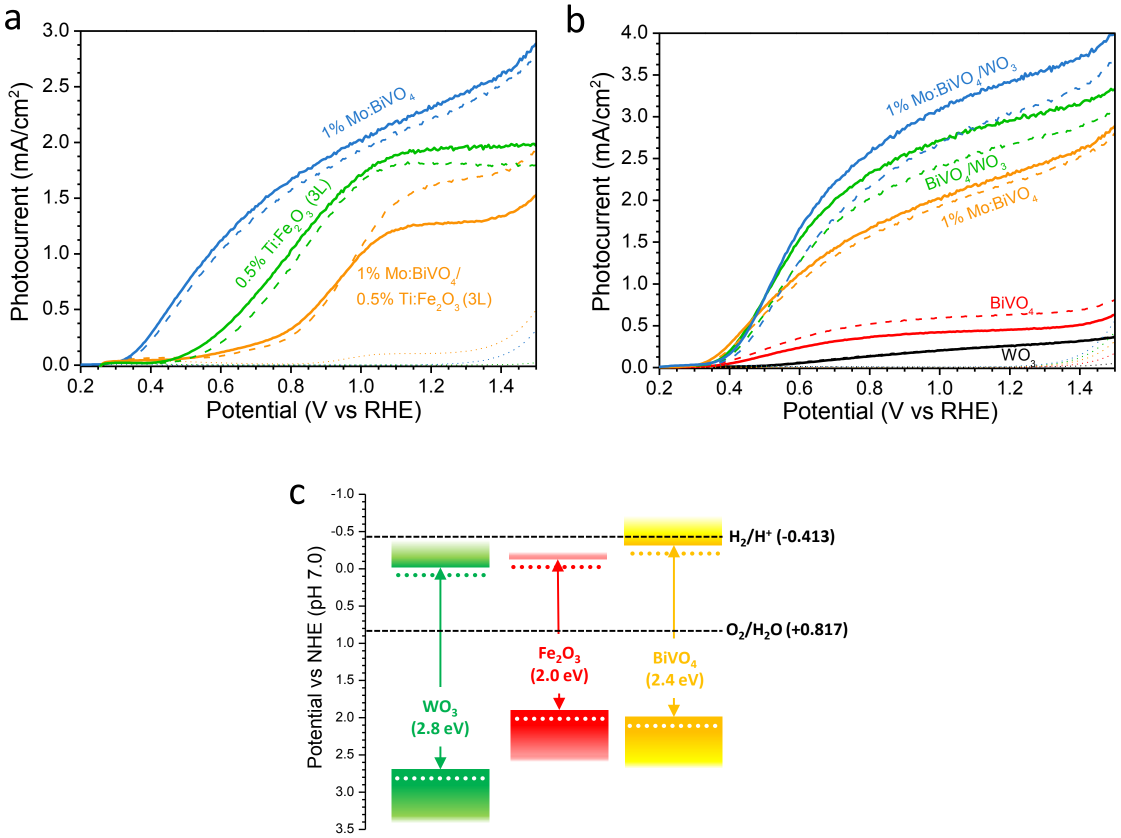

Supplementary Figure 1. I-V curves of bilayer-type heterojunction photoanodes. (a) 1% Mo:$BiVO_4$ (top)/0.5% Ti:Fe_2O_3. (b) 1% Mo:$BiVO_4$ (top)/WO_3 heterojunction photoanodes. Solid lines: I-V curves measured with front side illumination, segmented lines: I-V curves of back side illumination, dotted line: dark currents (Measurement conditions: AM 1.5 G filtered or 100 mW/cm^2, 0.5 M KPi + 0.5 M Na_2SO_3, scan rate: 20 mV/sec, forward scan). (c) Band alignments of WO_3, Fe_2O_3, and $BiVO_4$.

.

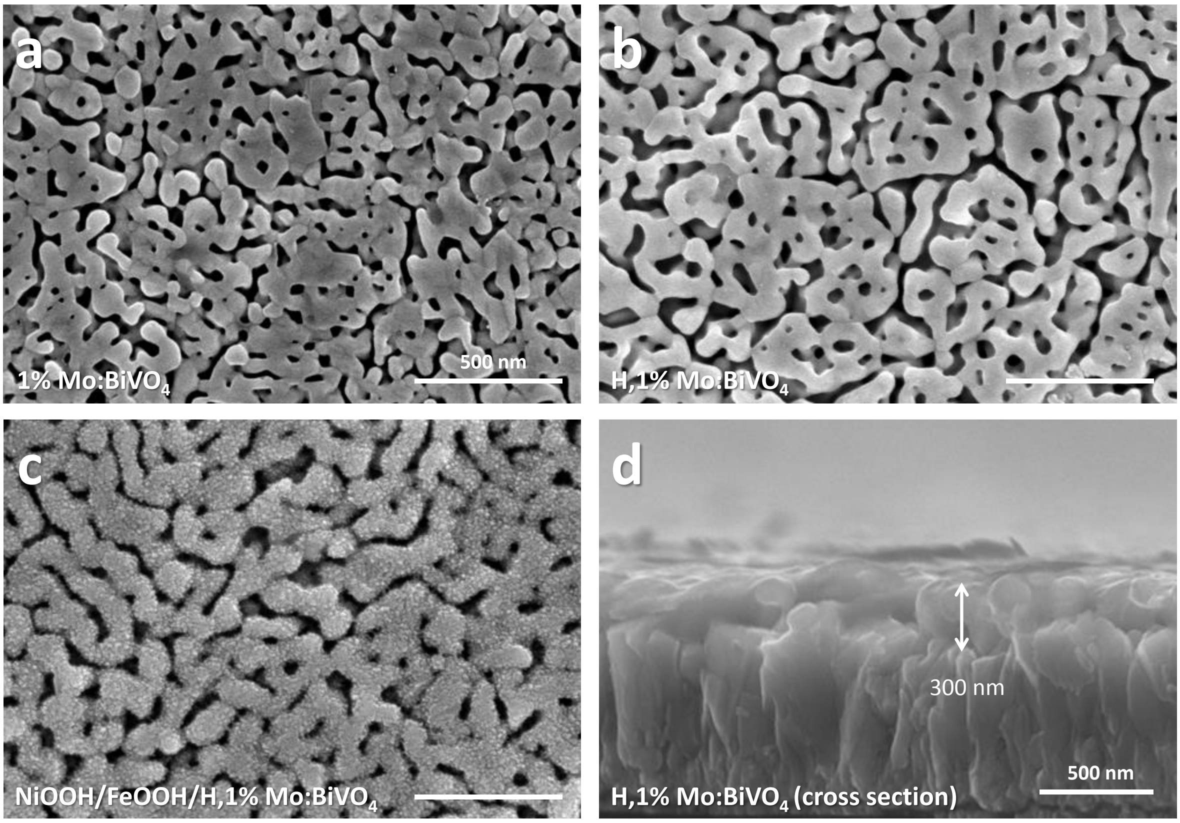

Supplementary Figure 2. Scanning electron micrographs of (a) 1% Mo-doped $BiVO_4$ (1% Mo:$BiVO_4$), (b) Hydrogen treated 1% Mo: $BiVO_4$ (H, 1% Mo: $BiVO_4$), and (c) NiOOH/FeOOH/H, 1% Mo: $BiVO_4$ (d) Cross-sectional image of H, 1% Mo: $BiVO_4$ photoelectrodes on FTO). Average film thickness is about 300 nm, and all scale bars are 500 nm.

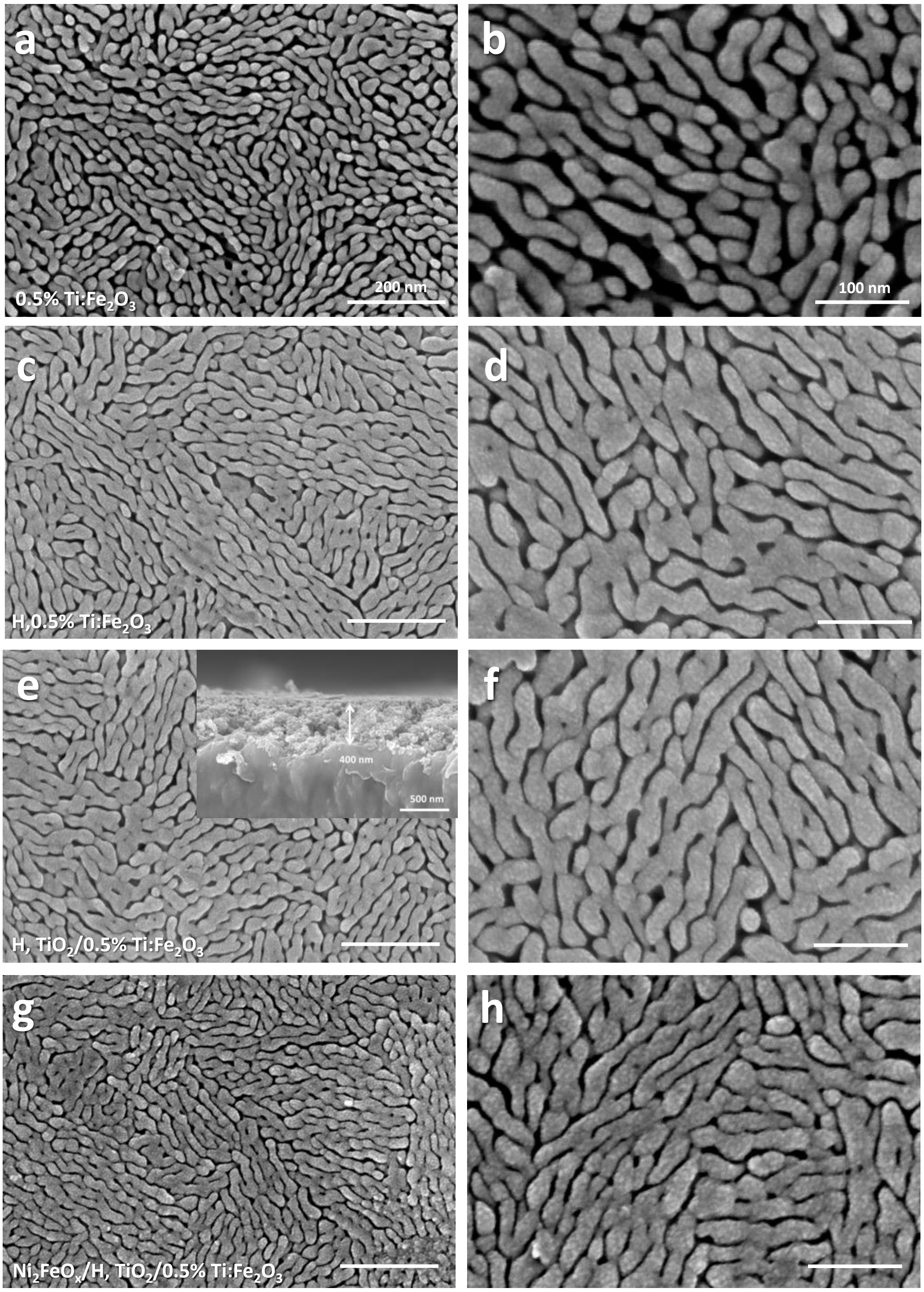

Supplementary Figure 3. Scanning electron micrographs of (a,b) 0.5% Ti-doped Fe_2O_3 (0.5% Ti: Fe_2O_3). (c,d) Hydrogen treated 0.5% Ti: Fe_2O_3 (H, 0.5% Ti: Fe_2O_3), (e,f) H, TiO_2/0.5% Ti: Fe_2O_3, and (g,h) $N_{i2}FeO_x$/H, TiO_2/0.5% Ti: Fe_2O_3. The scale bar of (a,c,e,g) are 200 nm and that of (b,d,f,h) are 100 nm. Inset in (e) shows cross-sectional image of H, TiO_2 /0.5% Ti: Fe_2O_3 on FTO with the thickness about 400~500nm.

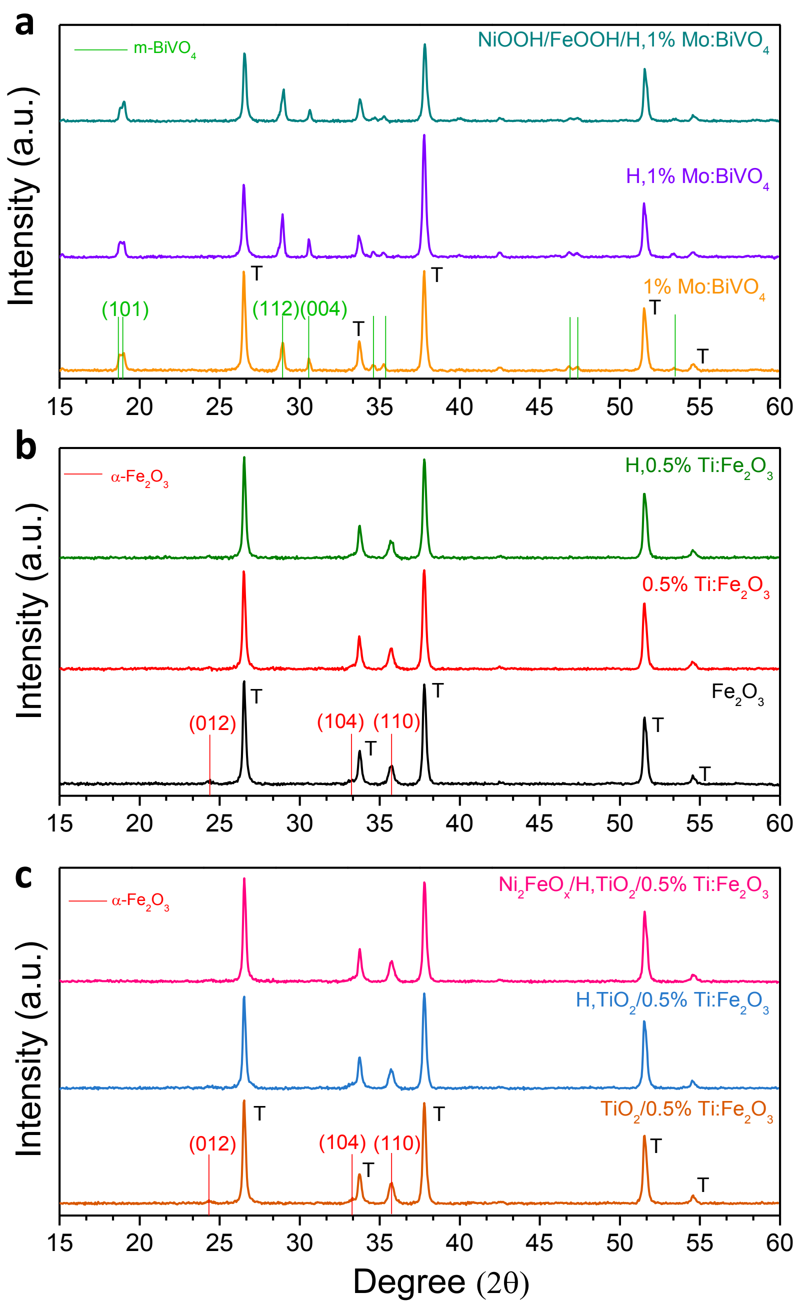

Supplementary Figure 4. XRD patterns of (a) $BiVO_4$-based photoanodes and (b-c) Fe_2O_3-based photoanodes. T represents patterns of FTO (F-doped tin oxide).

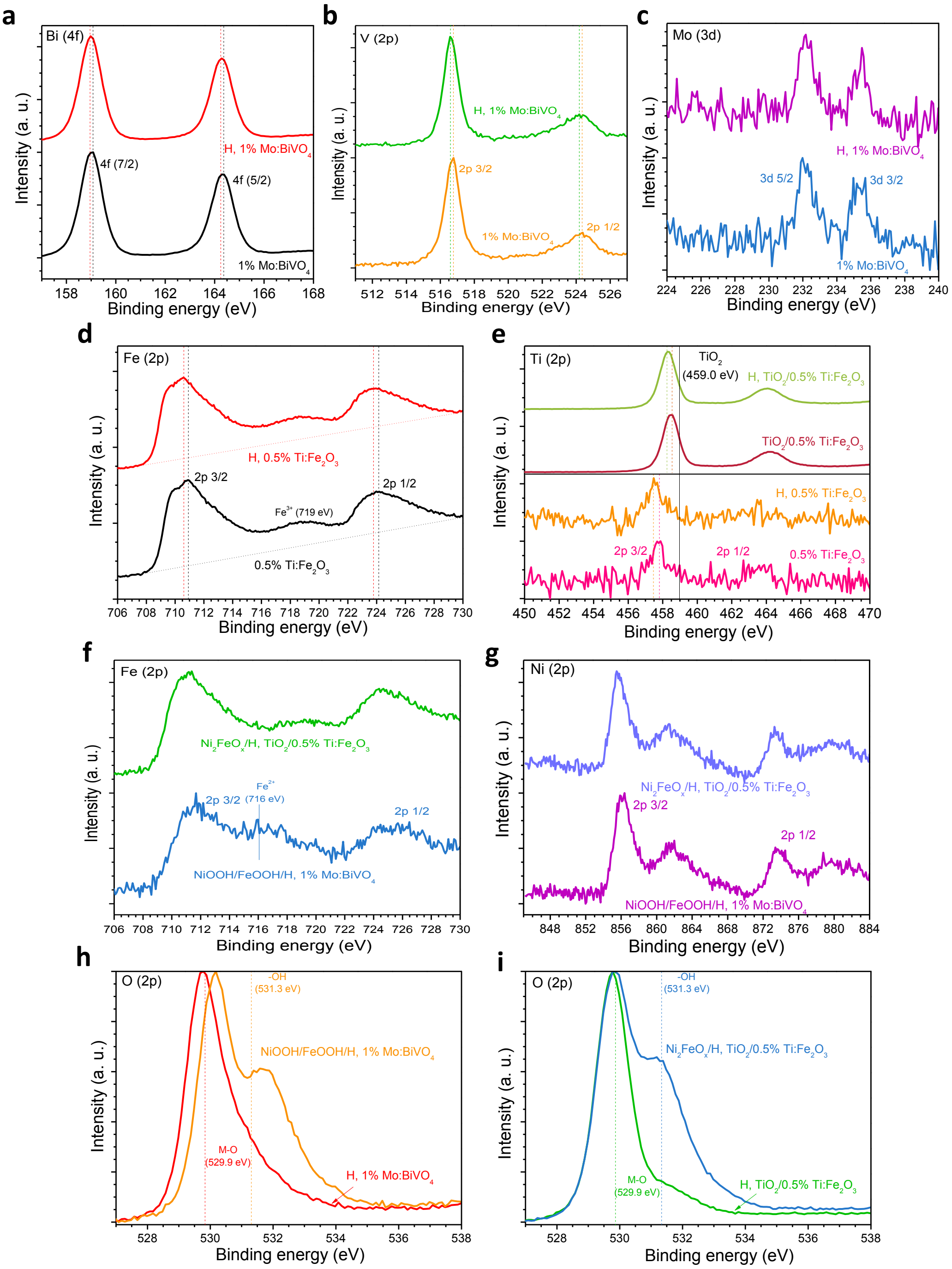

Supplementary Figure 5. X-ray photoelectron spectra (XPS) of (a) Bi 4f, (b) V 2p and (c) Mo 3d of 1% Mo:$BiVO_4$ and H, 1% Mo: $BiVO_4$. (d) Fe 2p, (e) Ti 2p of 0.5% Ti: Fe_2O_3, H, 0.5% Ti: Fe_2O_3, TiO_2/0.5% Ti: Fe_2O_3 and H, TiO_2/0.5% Ti: Fe_2O_3. (f) Fe 2p, (g) Ni 2p, (h, i) O 1s of Ni_2FeO_x/H, TiO_2/0.5% Ti: Fe_2O_3 and NiOOH/FeOOH/H, 1% Mo:$BiVO_4$ (All peak intensities have been normalized).

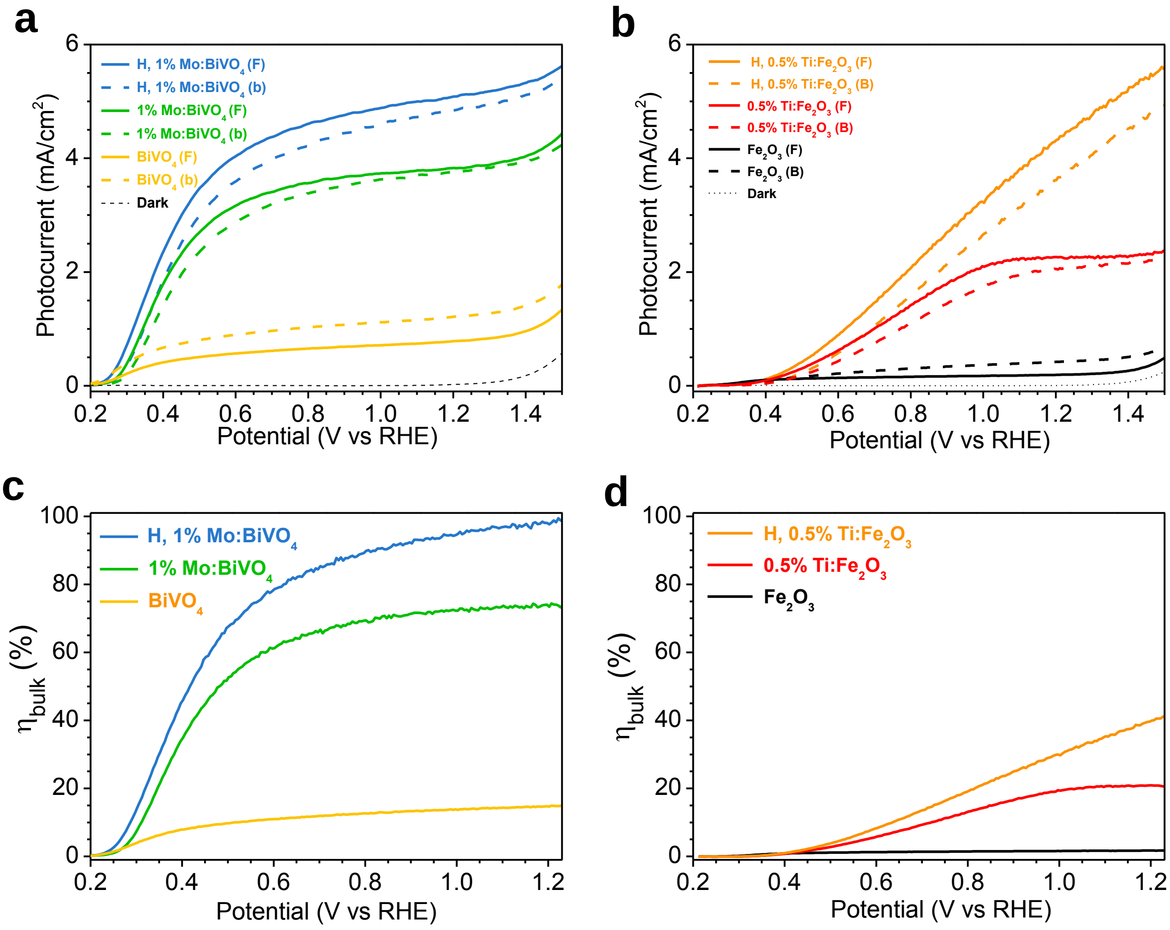

Supplementary Figure 6. I-V curves of photoanodes in the sulfite sacrificial reagent; (a) $BiVO_4$-based photoanodes, (b) Fe_2O_3-based photoanodes, and (c,d) bulk separation efficiency calculated with J_{abs} values of the materials. Measurement conditions: AM 1.5G (100 mW/cm^2), 0.5 M KPi + 0.5 M Na_2SO_3, pH 7.0, scan rate 20 mV/sec. F and B next to material denote front and back side illumination, respectively.

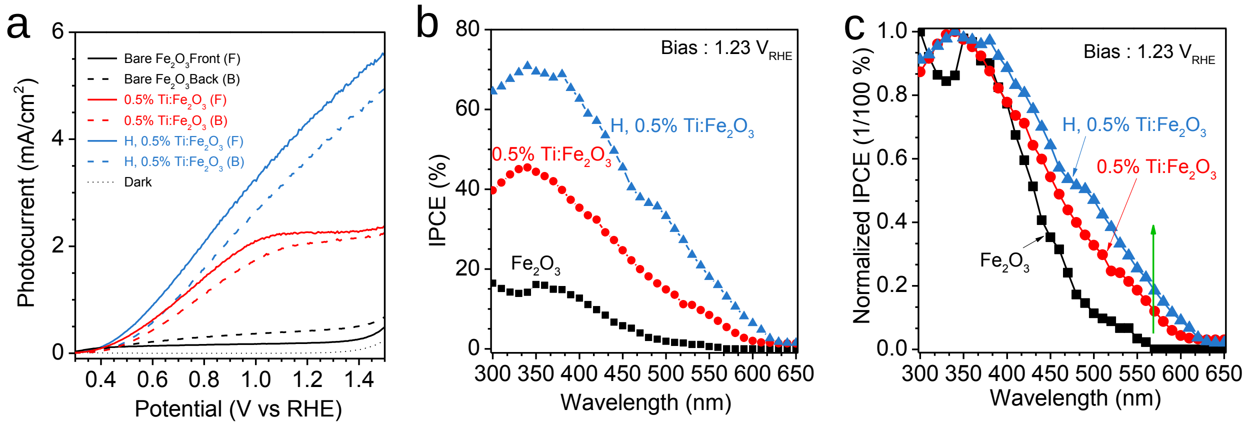

Supplementary Figure 7. (a) Steady-state current-potential (I-V) behaviors for Fe_2O_3 photoanodes. (b) Incident photon-to-current conversion efficiency (IPCE), (c) Normalized IPCE (0.5 M KPi and 0.5 M Na_2SO_3, pH = 7.0) of bare Fe_2O_3, 0.5% Ti: Fe_2O_3 and H, 0.5% Ti: Fe_2O_3.

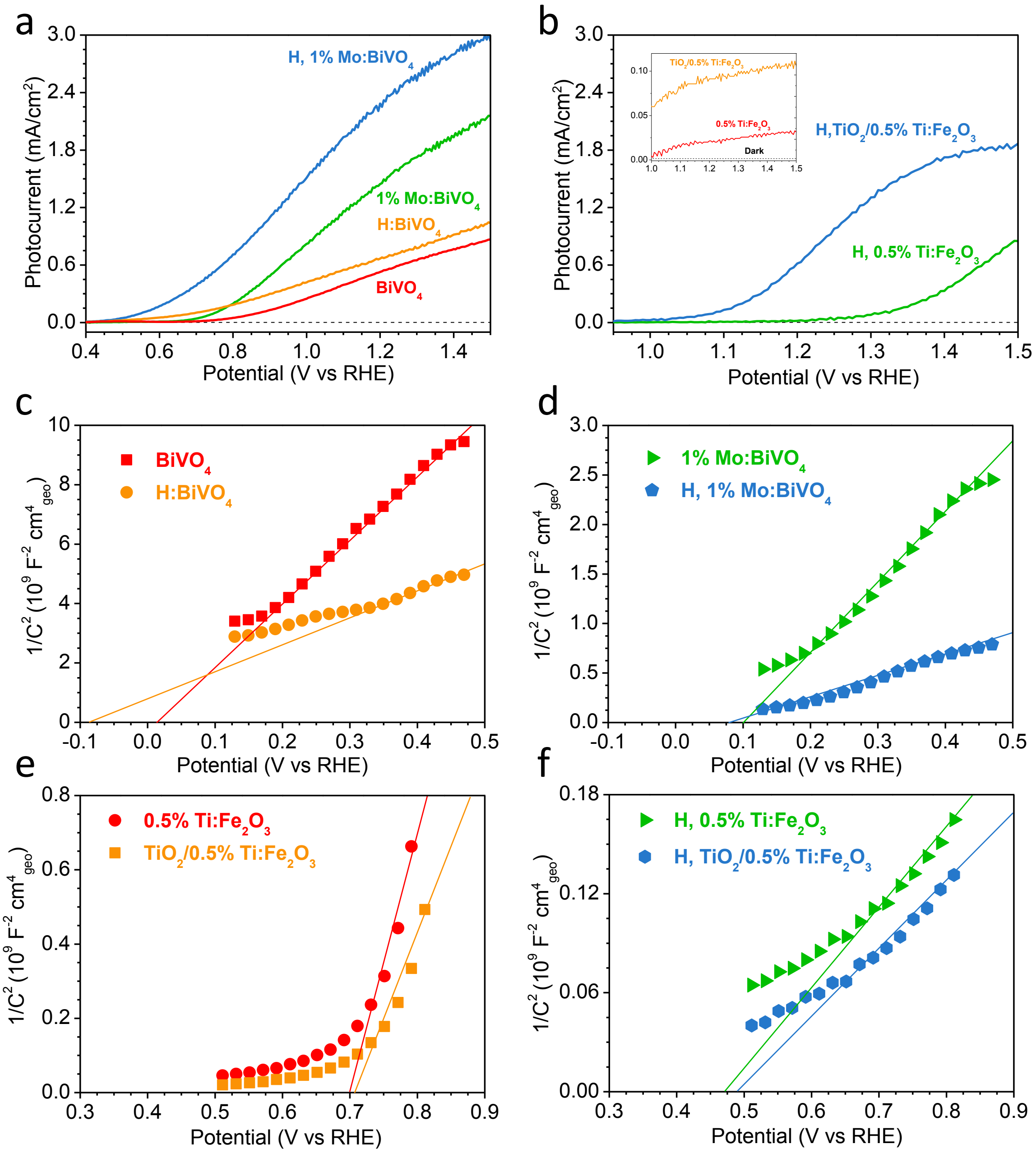

Supplementary Figure 8. I-V curve of photoanodes without cocatalysts (a, b) and Mott-Schottky plots: (c, d: $BiVO_4$ based photoanodes) and (e, f): Fe_2O_3-based photoanodes) measured in 1.0 M KCi (pH 9.2) under dark, with a frequency of 1000 Hz and scan rate: 20mV/sec. Dotted lines in (a) and (b): dark currents.

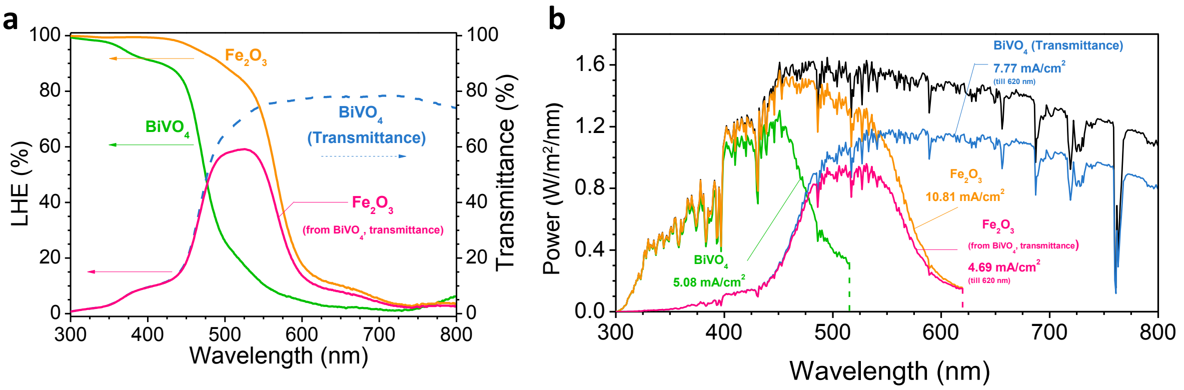

Supplementary Figure 9. (a) Light harvesting efficiency (LHE), transmittance (T) of $BiVO_4$ and Fe_2O_3, and light absorbance of Fe_2O_3 from transmitted light through $BiVO_4$. (b) LHE corresponding to AM 1.5G spectrum. Absorption photocurrents (J_{abs}): $BiVO_4$ (5.08 mA/cm^2, <516 nm), Fe_2O_3 (10.81 mA/cm^2, <620 nm), Fe_2O_3 behind $BiVO_4$ (4.69 mA/cm^2, <620 nm) and HDP ($BiVO_4$||Fe_2O_3) (5.08 mA/cm^2, <516 nm + 4.69 mA/cm^2, <620 nm = 9.77 mA/cm^2) calculated using light utilization threshold of 2.4 eV and 2.0 eV for $BiVO_4$ and Fe_2O_3. Samples used for analysis were H, 1% Mo:$BiVO_4$ and H, TiO_2/0.5% Ti:Fe_2O_3, respectively. Please note that absorption of $BiVO_4$ at >516 nm is not counted as available for HDP.

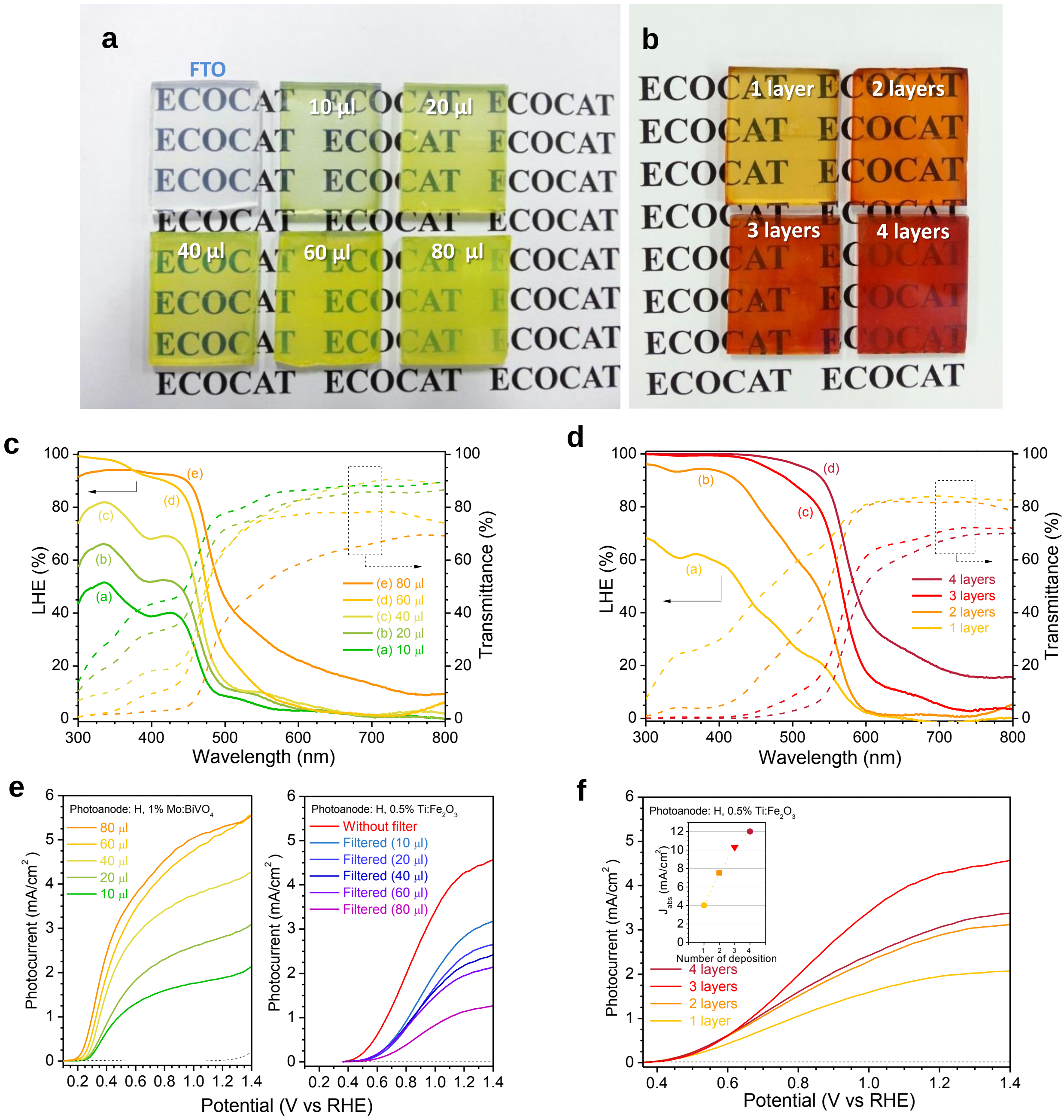

Supplementary Figure 10. Picture of (a) $BiVO_4$ and (b) Fe_2O_3 in different amounts of loading (used amounts of precursor for $BiVO_4$ and number of layers for Fe_2O_3). Optical property of (c) $BiVO_4$ and (d) Fe_2O_3. (e) I-V behaviors (sulfite oxidation) for $BiVO_4$ made with different amount of precursor (left) and corresponding I-V curve of Fe_2O_3 photoanode under such front light absorbers (right, denoted as filtered). (f) I-V curve of Fe_2O_3 photoanodes with different number of loading (inset graph is for Jabs current calculated from above (d)). Denotation for $BiVO_4$ - H, 1% Mo:$BiVO_4$ and Fe_2O_3 – H, 0.5% Ti:Fe_2O_3 was used. Measurement was conducted in 0.5 M Na_2SO_3 + 0.5 M KPi, pH 7.0 with AM 1.5G illumination (100 mW/cm^2). Dotted lines in (e) and (f): dark currents.

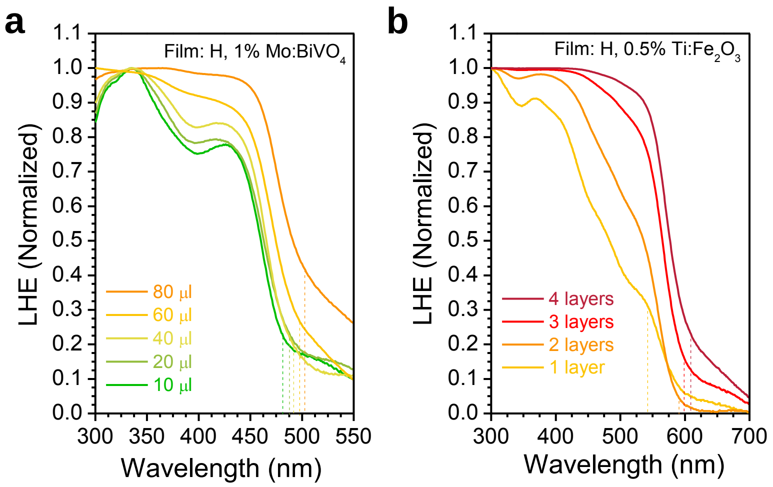

Supplementary Figure 11. Normalized LHE of (a) $BiVO_4$ and (b) Fe_2O_3 with different amount of deposition. Dotted line was drawn at the point of curvature intersection. Denotation for $BiVO_4$ - H, 1% Mo:$BiVO_4$ and Fe_2O_3 – H, 0.5% Ti:Fe_2O_3 was used.Optimization of $BiVO_4$ and Fe_2O_3 used in this study was conducted for both optimal performance for individual photoanode and HDP. Very obviously, lower amount of deposition granted larger transmittance but smaller LHE for itself and higher amount of deposition gave result with opposite tendency. Increasing amount of $BiVO_4$ deposition granted progressively higher performance for HDP till transmittance of $BiVO_4$ was compromised at deposition amount of 80 µl per 5.0 cm^2. (Fig. 1D). While the highest photocurrent for $BiVO_4$ came from 80 µl per 5.0 cm^2 (5.40 mA/cm^2 at 1.23 V_{RHE}), significantly deterred transmittance resulted significantly lower photons for underlying Fe_2O_3.

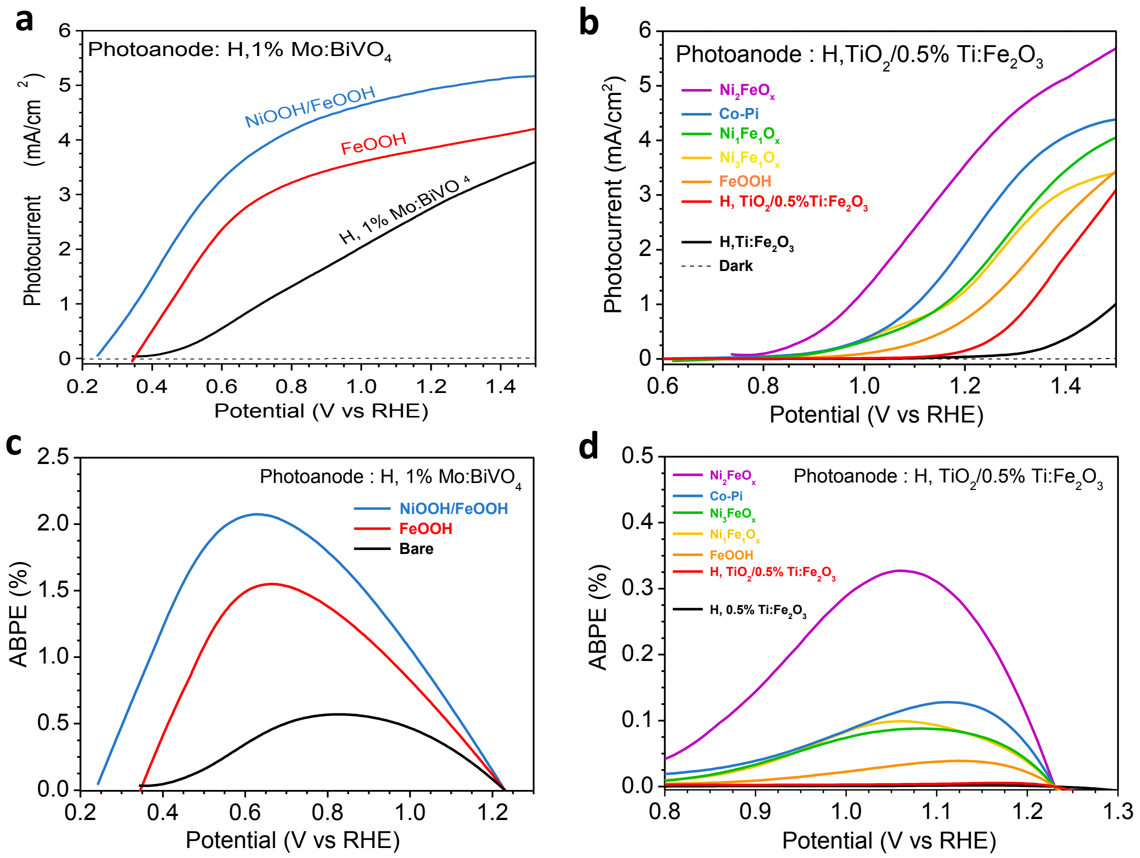

Supplementary Figure 12. Photocurrent generation and applied bias photon-to-current efficiency (**ABPE**) for PEC water splitting with various co-catalysts on (a,c) H,1% Mo: $BiVO_4$ and (b,d) H,TiO_2/0.5% Ti: Fe_2O_3. (electrolyte: 1.0 M KCi, pH = 9.2). Dotted lines in (a) and (b): dark currents.

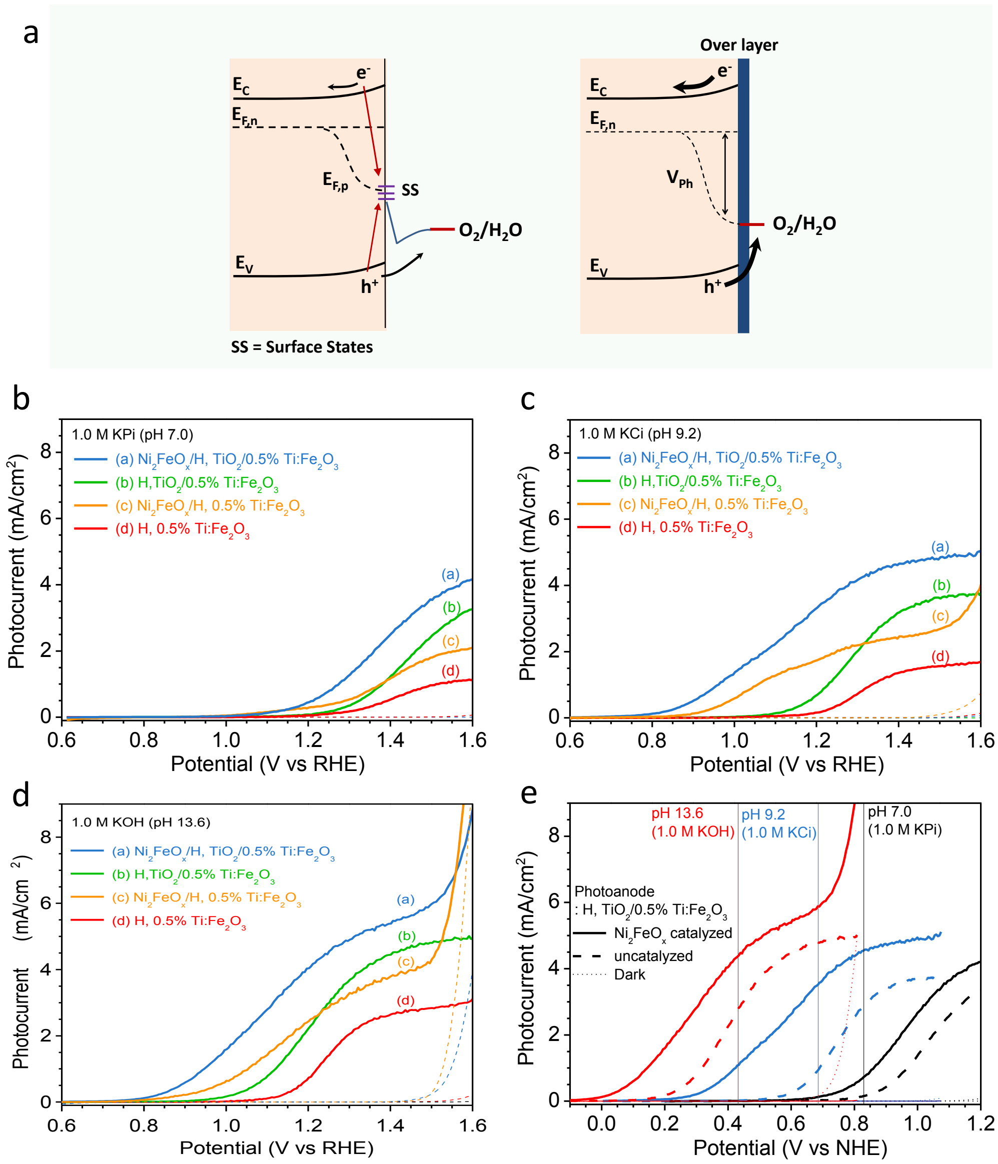

Supplementary Figure 13. Effects of surface passivation by overlayer and electrolyte pH for Fe_2O_3 based photoanodes**.** (a) Schematics showing the effect of surface states and passivation by an overlayer. (b) 1.0 M KPi, pH 7.0, (c) 1.0 M KCi, pH 9.2 and (d) 1.0 M KOH, pH 13.6. (e) I-V curves of Ni_2FeO_x catalyzed and uncatalyzed H, TiO_2/0.5% Ti:Fe_2O_3. Vertical line of red (1.0 M KOH), blue (1.0 M KCi) and black (1.0 M KPi) presents position of 1.23 V_{RHE} with conversion of NHE potential scale. Dotted lines in (a),(b) and (c) represent dark currents of photoanodes.

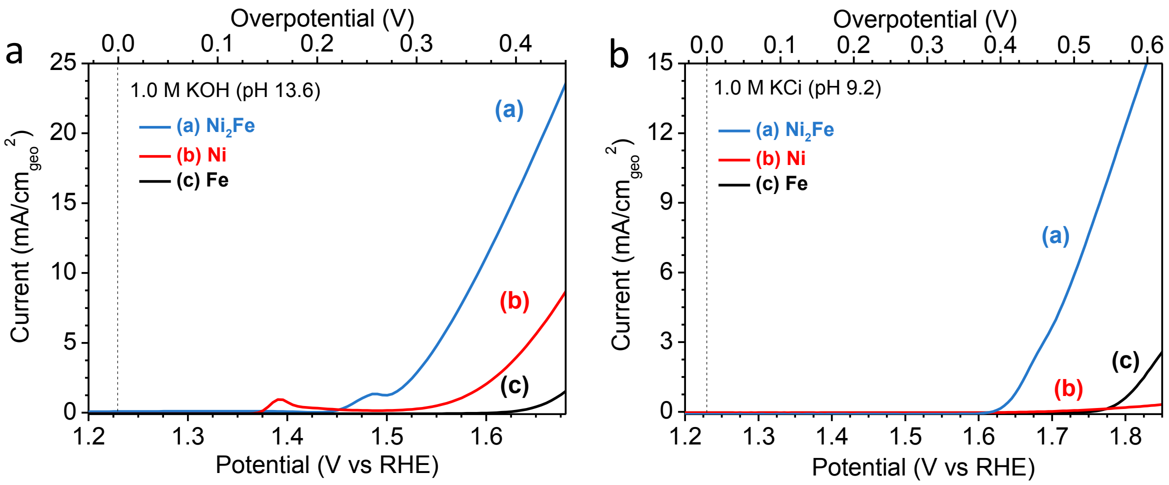

Supplementary Figure 14. Effects of pH of electrolyte and for Ni_2FeO_x, FeO_x and NiO_x electrocatalysts on FTO. (a) 1.0 M KOH (pH 13.6) and (b) 1.0 M KCi (pH 9.2). Similar process was used for electrocatalyst deposition. Thus, Ni, Fe and Ni_2Fe precursors (0.2 M, 3 ul for each) were dropped on FTO glass (0.5 cm^2) and dried for 30 seconds in fume hood. Then samples were gently rinsed with 1.0 M KOH for 10 seconds before analysis. And sample was directly used for electrochemical analysis. Scan rate: 20 mV/second.

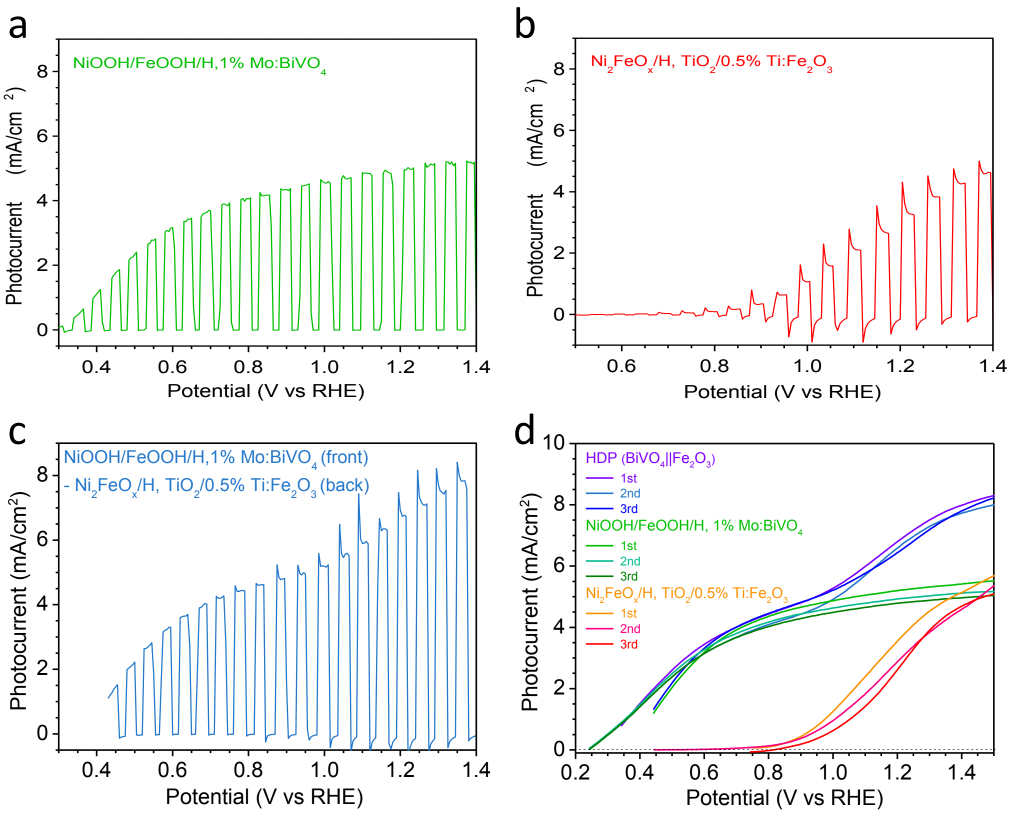

Supplementary Figure 15. Chopped illumination used I-V curves of (a) NiOOH/FeOOH/H, 1% Mo:$BiVO_4$, (b) Ni_2FeO_x/H, TiO_2/0.5% Ti:Fe_2O_3, (c) HDP (NiOOH/FeOOH/H, 1% Mo:$BiVO_4$|| Ni_2FeO_x/H, TiO_2/0.5% Ti:Fe_2O_3) and (d) best results of each photoelectrode (electrolyte : 1.0 M KCi, pH = 9.2). Photoanodes used for analysis recorded 4.5~5.0 mA/cm^2 for $BiVO_4$, 3.3~4.1 mA/cm^2 for Fe_2O_3 and 6.5~7.0 mA/cm^2 for HDP at 1.23 V_{RHE}.

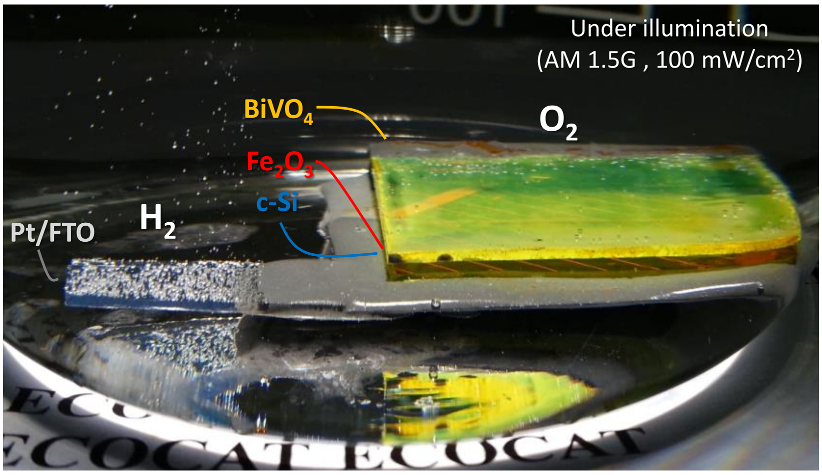

Supplementary Figure 16. Artificial leaf (monolithic tandem cell) in action under simulated 1 sun condition. The artificial leaf was made with HDP ($BiVO_4$||Fe_2O_3) – 2p c-Si solar cell. Measurement conditions: AM 1.5G (100 mW/cm^2), 1.0 M KCi (pH 9.2), active area: 5.0 cm^2, counter electrode area: 1.0 cm^2). See **Supplementary Movie 2.**.

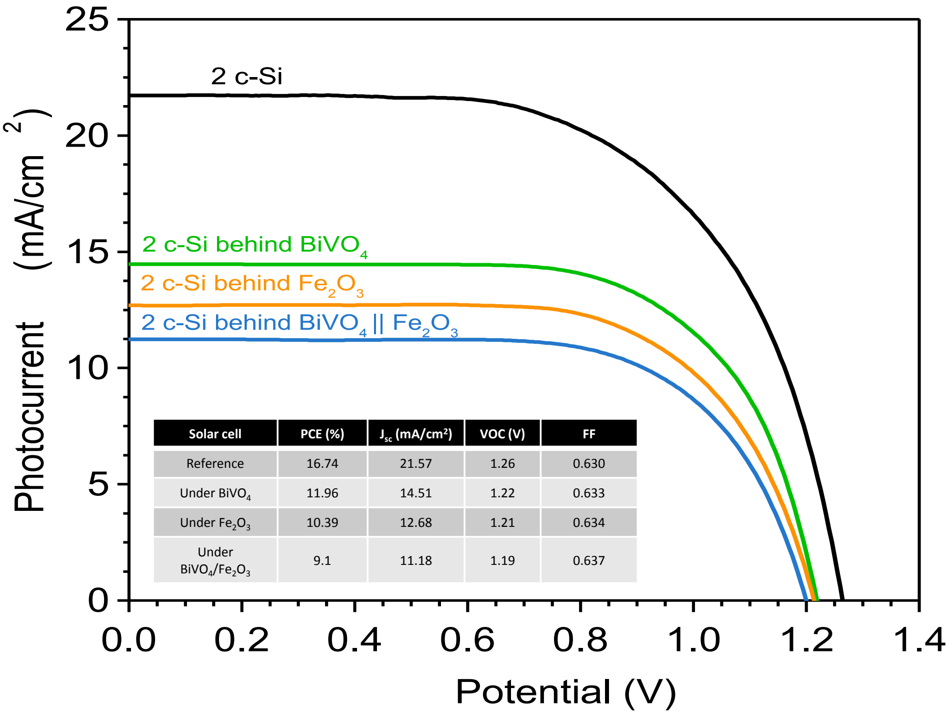

Solar cell	PCE (%)	J_{sc} (mA/cm²)	VOC (V)	FF
Reference	16.74	21.57	1.26	0.630
Under $BiVO_4$	11.96	14.51	1.22	0.633
Under Fe_2O_3	10.39	12.68	1.21	0.634
Under $BiVO_4/Fe_2O_3$	9.1	11.18	1.19	0.637

Supplementary Figure 17. I-V curves of 2p c-Si solar cell with/without $BiVO_4$, Fe_2O_3 and $BiVO_4||Fe_2O_3$ films as 1st absorber under 1 sun (AM 1.5G). Inset table contains performance data of 2p c-Si solar cell with active area of 0.25 cm^2.

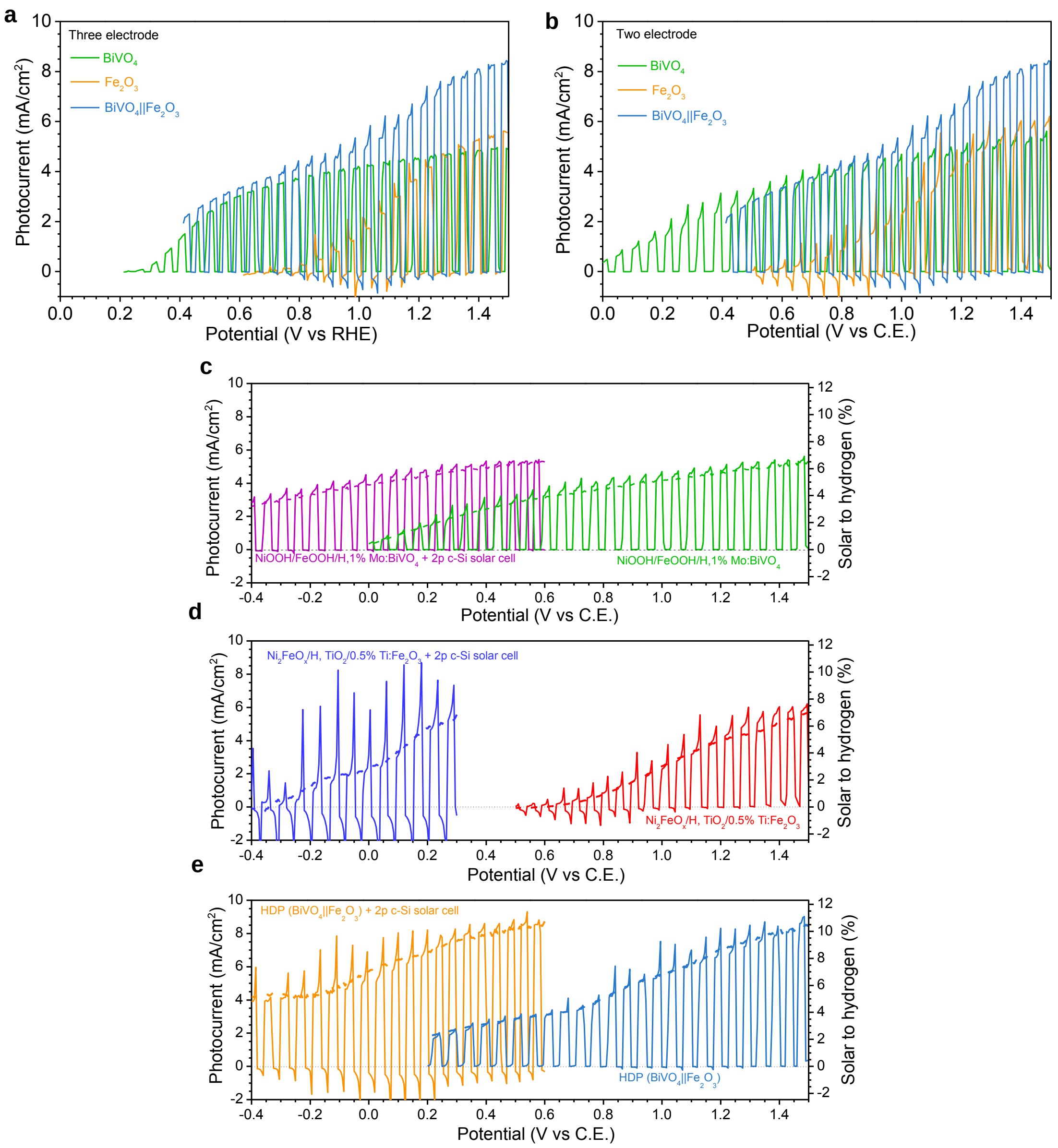

Supplementary Figure 18. I-V curves of photoanodes with (a) three and (b) two electrode configuration for measurements. I-V curve of actual tandem cell composed with metal oxide film/2 c-Si solar cell are compared with corresponding photoanodes - (c) NiOOH/FeOOH/H, 1% Mo:$BiVO_4$, (d) Ni_2FeO_x/H, TiO_2/0.5% Ti:Fe_2O_3 and (e) HDP($BiVO_4$||Fe_2O_3). Measurement conditions: AM 1.5G (100 mW/cm^2), 1.0 M KCi (pH 9.2), backward scan (20 mV/sec), Pt mesh as the counter electrode, active area: 0.24 cm^2).

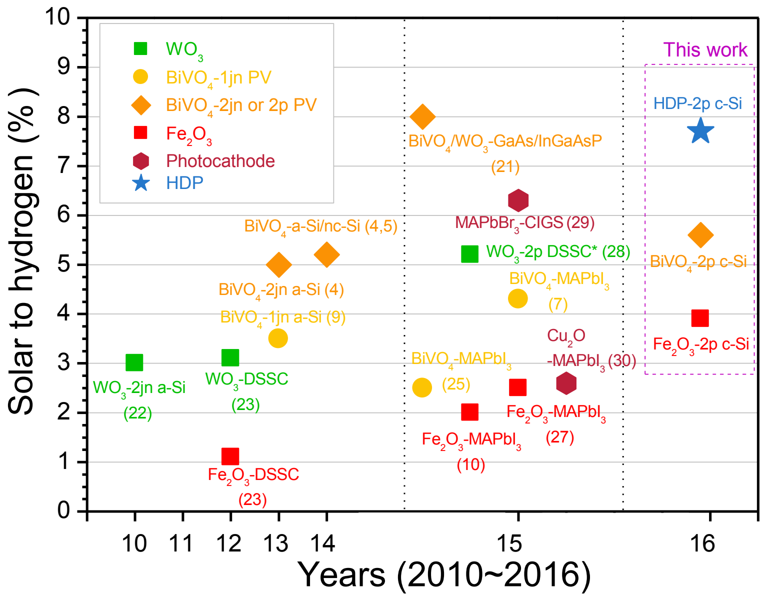

Supplementary Figure 19. Performance records of photoanode – photovoltaic solar cell tandem cells (D4~T6). WO_3, Fe_2O_3, $BiVO_4$ are selected as representative absorbers while recent photocathode related reports (CIGS, Cu_2O) were also included owing to their characteristics as photoelectrode. Order is in arrangement of 1st/2nd/3rd absorbers and number marked at last is reference number. Detailed data are given in Supplementary Table S2.

Supplementary Table 1. Reported photocurrents of metal oxide photoanodes.

Year	Photoanode	Measurement conditions	Performance & features	Publication Institute(s)
2003	$BiVO_4$	0.5 M Na_2SO_4 (pH = 6.6) Xe lamp (500 W) with/without band pass filters	~0.8mA/cm^2<br>(0.4 V vs. Ag/AgCl)<br>~29 % IPCE at 420 nm (1.3 V vs. Ag/AgCl)	Sayama *et al.*[1]<br>National Institute of Advanced Industrial Science and Technology (AIST)
2010	$BiVO_4$	1.0 M KCi<br>(CO_2 Saturated, pH 7.8)<br>100 mW/cm^2	1.59 mA/cm^2 (1.23 V_{RHE})<br>~32% IPCE at 420 nm<br>(1.23 V_{RHE})	Sayama *et al.*[2]<br>AIST
2011	RhO_2/Mo:$BiVO_4$	Sea water<br>100 mW/cm^2	2.7 mA/cm^2 (1.23 V_{RHE})<br>~50% IPCE at 365~450 nm<br>(1.0 V_{RHE})	*Luo et al.*[3]<br>Nanjing University (NJU)
2013	Co-Pi/W:$BiVO_4$	0.1 M KPi (pH 7)<br>100 mW/cm^2	3.60 mA/cm^2 (1.23 V_{RHE})<br>**T6 tandem cell with 2jn a-Si<br>(STH : 5.0 %)	Abdi *et al.*[4]<br>Delft University of (Delft) Technology / Helmholtz Zentrum Belin (HZB)
2014	Co-Pi/W:$BiVO_4$	0.1 M KPi (pH 7)<br>100 mW/cm^2	4.00 mA/cm^2 (1.23 V_{RHE})<br>T6 tandem cell with 2jn a-Si<br>(STH : 5.2 %)	Han *et al.*[5]<br>Delft/ HZB
2014	NiOOH/FeOOH/$BiVO_4$	0.5 M KPi (pH 7)<br>100 mW/cm^2	~4.3 mA/cm^2 (1.23 V_{RHE})<br>~60 % IPCE at 420 nm (0.6 V_{RHE})<br>Stable for +50 hours<br>ABPE* ~1.8%	Kim *et al*[6]<br>University of Wisconsin–Madison (UW-Madison)
2015	Co-Ci/H_2 treated Mo:$BiVO_4$	0.1 M KCi (pH 7)<br>100 mW/cm^2	4.8 mA /cm^2 (1.23V_{RHE})<br>Stable for +12 hours<br>D4 tandem cell with $MAPbI_3$<br>(STH : 4.8%)	Kim *et al.*[7]<br>Ulsan National Institute of Science and Technology (UNIST) / Pohang University of Science and Technology (POSTECH)
2015	NiOOH/FeOOH<br>/N_2-treated $BiVO_4$	0.5 M KPi (pH 7)<br>100 mW/cm^2	~5.0 mA /cm^2 (1.23V_{RHE})<br>Stable for +30 hours<br>ABPE ~2.2%	Kim *et al.*[8]<br>University of Wisconsin–Madison (UW-Madison)
**Present**	**NiOOH/FeOOH<br>/H_2 treated Mo: $BiVO_4$**	**1.0 M KCi<br>(pH 9.2)<br>100 mW/cm^2**	**5.0 mA/cm^2 (1.23 V_{RHE})<br>~86% IPCE at 420 nm<br>(1.23 V_{RHE})**	**This work**
2000	Fe_2O_3 Nanorods	0.1 M KI (pH 6.8)	~1μA/cm^2 (0.3 V *vs.* Ag/AgCl)	Beermannt *et al.*[9]<br>Uppsala University (UU)
2005	Fe_2O_3<br>(ultrasonic spray pyrolysis)	1.0 M NaOH<br>100 mW/cm^2	1.5 mA/cm^2 (1.23 V_{RHE})<br>14 % IPCE at 400 nm<br>(1.23 V_{RHE})	Duret *et al.*[10]<br>École polytechnique fédérale de Lausanne (EPFL)
2010	IrO_2 /Cauliflower<br>-type Si:Fe_2O_3	1.0 M NaOH<br>100 mW/cm^2	3.3 mA/cm^2 (1.23 V_{RHE})<br>50% IPCE at 320 nm<br>(1.23 V_{RHE})	Tilley *et al.*[11]<br>EPFL
2013	Co-Pi/Pt:Fe_2O_3	1.0 M NaOH<br>100 mW/cm^2	4.32 mA/cm^2 (1.23 V_{RHE})	Kim *et al*[12]<br>POSTECH
**Present**	**Ni_2FeO_x/H_2 treated TiO_2/<br>Ti doped Fe_2O_3**	**1.0 M KCi or<br>1.0 M KOH<br>100mW/cm^2**	**~4.50 mA/cm^2 (1.23 V_{RHE})<br>50% IPCE at 320 nm**	**This work**
2001	Nanoporous WO_3	1 M $HClO_4$<br>100 mW/cm^2	2.4 mA/cm^2 (1.23 V_{RHE})<br>75% IPCE at 410nm<br>(1.0 V_{RHE})	Santato *et al.*[13]<br>University of Geneva (UNIGE)
2008	Nanoporous WO_3	3 M H_2SO_4 / 0.5 M NaCl<br>100 mW/cm^2	3.0 mA/cm^2 (1.23 V_{RHE})<br>80 % IPCE at 410nm<br>(1.0 V_{RHE})	Alexander *et al.* [14]<br>University of Warsaw
2010	Nanoporous WO_3	0.5 M H_2SO_4<br>100mW/cm^2	3.45 mA/cm^2<br>(1.6 V *vs.* Ag/AgCl)<br>92% IPCE at 340nm<br>(1.2 V *vs.* Ag/AgCl)	Li *et al.* [15]<br>Central South University (CSU)
2011	Mesoporous WO_3	100 mW/cm^2	3.7 mA/cm^2<br>(1.3 V *vs.* Ag/AgCl)	Kim *et al.*[16]<br>Sungkyunkwan University (SKKU)
2011	$BiVO_4$/WO_3 nanorods	0.5 M Na_2SO_4<br>100 mW/cm^2	0.8 mA/cm^2 (1.0 V vs. NHE)<br>31% IPCE at<br>(1.0 V vs. Counter electrode)	Su *et al.*[17]<br>Xi'an Jiaotong University(XJTU) / Nanjing University of Technology (NUST)
2011	$BiVO_4$/WO_3	0.5 M Na_2SO_4<br>100 mW/cm^2	1.74 mA/cm^2 (1.6 V *vs.* Ag/AgCl)	Hong *et al.*[18]<br>POSTECH
2012	$BiVO_4$/SnO_2/WO_3	Saturated KCi (pH 7)<br>100 mW/cm^2	3.0 mA/cm^2 (1.23 V_{RHE})<br>Dual: 4.10 mA/cm^2 (1.23 V_{RHE})	Saito *et al.*[19]<br>AIST
2014	NiOOH/FeOOH/W,<br>Mo:$BiVO_4$/helix WO_3	0.5 M K_2SO_4 + buffer<br>(pH 7)<br>100 mW/cm^2	5.34 mA/cm^2 (1.23 V_{RHE})<br>~90% IPCE at 420 nm (1.23 V_{RHE})	Shi *et al.*[20]<br>SKKU
2015	Co-Pi/$BiVO_4$/nano wire WO_3	Phosphate buffer<br>(pH 7)<br>100 mW/cm^2	6.72 mA/cm^2 (1.23 V_{RHE})<br>~30% IPCE at 530 nm (1.23 V_{RHE})<br>T6 tandem cell with III-V Ga, In, As, P based solar cell<br>(STH 8.0%)	Pihosh *et al.*[21]<br>University of Tokyo (UTokyo)<br>/ AIST
**Present**	**HDP($BiVO_4$||Fe_2O_3)<br>NiOOH/FeOOH/H_2 treated Mo doped $BiVO_4$<br>Ni_2FeO_x/ H_2 treated TiO_2/Ti doped Fe_2O_3**	**1.0 M KCi (pH 9.2)<br>100 mW /cm^2**	**HDP -7.0 mA/cm^2 (1.23 V_{RHE})<br>~95% IPCE at 450 nm<br>~20% IPCE at 530 nm<br>(HDP, 1.23V_{RHE})**<br>Q6 tandem cell with 2p c-Si (STH 7.7 %)	**This work**

*KPi: potassium phosphate, KCi: potassium bicarbonate, KOH: potassium hydroxide.
**T6: triple light absorbers for 6 photons for 1 molecule of hydrogen evolution, D4: double light absorbers / 4 photons, Q6: quadruple light absorbers /6 photons.

Supplementary Table 2. Recent reports on highly active unbiased solar water splitting by photoelectrode-photovoltaic combination (2010~2016).

years	*OEC/light absorbers/HEC	**No. of light absorber	Electrolyte	Efficiency (STH)	***Gas evolution & Stability	Ref
2010	RuO_2/WO_3-2jn a-Si/NiMo	3	0.33 M H_3PO_4	3%		22
2012	Fe_2O_3-DSSC/Pt<br>WO_3-DSSC/Pt	2	1 M NaOH (pH 13.6)<br>1 M $HClO_4$ (pH 0)	Fe_2O_3 - 1.1%<br>WO_3 - 3.1 %	OER/HER (8 hours)	23
2014	FeOOH/Mo:$BiVO_4$-3jn a-Si/Ni	4	0.5 M NaPi (pH 7)	2.5%	OER/HER (12 hours)	24
2013,2014	Co-Pi/W:$BiVO_4$-1,2jn a-Si/Pt	2 (1jn a-Si)<br>3 (2jn a-Si)	0.1 M KPi (pH 7)	3.5% (1jn)<br>5.0% (2jn)	(~1 hours)	4,5
2015	Co-Pi/$BiVO_4$-$MAPbI_3$/Pt	2	0.1 M phosphate buffer (pH 7)	2.5%	HER in sacrificial reagent	25
2015	Co-Pi/Mn:Fe_2O_3-$MAPbI_3$/Pt	2	1 M NaOH (pH 13.6)	2.4%	OER/HER (8 hours)	26
2015	$NiFeO_x/Al_2O_3$/Si:Fe_2O_3-$MAPbI_3$/NiMo	2	1 M NaOH (pH 13.6)	2.0%	OER/HER (8 hours)	27
2015	Co-Pi/$BiVO_4/WO_3$-GaAs/InGaAsP/InP/Pt	3	Phosphate buffer (pH 7)	8.0%	OER/HER (1 hours)	21
2015	Co-Ci/H,Mo:$BiVO_4$-$MAPbI_3$/Pt	2	0.1 M KCi (pH 7)	4.3% (wire)<br>3% (monolithic)	OER/HER (12 hours)	7
2015	WO_3 – 2p DSSC/Pt	3	1.0 M H_2SO_4	5.2%**** ($S_2O_8^{2}$/H_2)<br>3.05% (O_2/H_2)	Peroxide production ($S_2O_8^{2-}$) (2 hours)	28
2015	Ir, Ru/Ti-$MAPbX_3$/CIGS/CdS/ZnO/TiO_2/Pt (X – Br, I)	2	0.5 M H_2SO_4 (pH 0)	2.6% ($MAPbI_3$)<br>6.3% ($MAPbBr_3$)	HER for photocathode (~ 1hr)	29
2015	IrO_2/$MAPbI_3$-Cu_2O/AZO/TiO_2/RuO_2	2	0.5 M Na_2SO_4 (buffered to pH 5.0)	2.6%	OER/HER (3 hours)	30
2016	NiOOH/FeOOH/H, Mo:$BiVO_4$ – Ni_2FeO_x/H, TiO_2/Ti:Fe_2O_3-2p c-Si/Pt	3	1.0 M KCi (pH 9.2)	5.6% ($BiVO_4$)<br>3.9% (Fe_2O_3)<br>7.7% (HDP)	OER/HER (8 hours)	This work

PA (photoanode) – PV is type that photoanode is electrically connected with PV and PV is also connected to counter electrode. STH value is recorded from operating point prediction or actual operation of tandem cell (higher value in report is taken).
*OEC: oxygen evolution catalyst, HEC: hydrogen evolution catalyst.
** Number of light absorbers is accessed by counting any semiconductors used for tandem cell. For example, Co-Pi/$BiVO_4$/2jn a-Si/Pt has one metal oxide absorber + two silicone absorbers, so counted as 3 (T6 system, triple absorber – 6 photons for 1 hydrogen molecule production).
2p – parallel solar cell, 2jn – double junction, 1jn single junction (if it is not denoted individually, it is 1jn).
Using two photoanodes is counted as one because of same hole/electron transfer passway.
*** Stability and gas evolution test for individual component or whole tandem cell are accounted.
****Efficiency calculated from peroxide – HER potential (2.12 V) instead of OER-HER (1.23 V), while unbiased photocurrent was 2.48 mA/cm^2. For actual energy used for hydrogen production, it is STH 3.05%.

Supplementary discussion

Calculation of surface/bulk charge separation efficiency

For quantitative assessment of charge separation efficiency, photocurrent comparison between water oxidation/hole scavengers (H_2O_2, SO_3^{2-}) was used.

Water oxidation: $2H_2O + 4h^+ \rightarrow 4H^+ + O_2$, $E^o = 1.23\ V_{RHE}$

Sulfite oxidation: $SO_3^{2-} + h^+ \rightarrow SO_3^-$, $E^o = 0.73\ V_{RHE}$

Light absorption by a photocatalyst generates absorbed photocurrent (J_{abs}) that undergoes two major losses of bulk and surface recombination. Hence the measured photocurrent during water oxidation (J^{H2O}) is expressed by;

$$J^{H2O} = J_{abs} \times \eta_{bulk} \times \eta_{surf} \quad (1)$$

where η denotes the charge separation yield in the bulk of semiconductor (η_{bulk}) or on the surface (η_{surf}). Since the surface charge separation yield of SO_3^{2-} is almost 100% ($\eta_{surf} = 1$) as discussed above, the photocurrent from its oxidation can be expressed as follows:

$$J^{SO3} = J_{abs} \times \eta_{bulk} \quad (2)$$

For calculation of J_{abs}, correlation below between absorbance and radiation proposed by Choi's group [1].

$$P_d = P_0 10^{-A} \quad (3)$$

$$P_{abs} = P_0(1-10^{-A}) \quad (4)$$

P_0 ($mWcm^{-2}nm^{-1}$) is provided power by solar simulator (in this case, AM 1.5G), P_{abs} is power of light actually absorbed by photoanode and P_d is power of light dissipated by reflection and penetration. A is absorbance of photoanode (in this case, $BiVO_4$, Fe_2O_3) and LHE (light harvesting efficiency) is defined as $1-10^{-A}$. So light which is not absorbed at photoanode will be 10^{-A}. Integrated $P_{abs}(\lambda)$ ($mWcm^{-2}nm^{-1}$) along with wavelength λ gives total power (unit of $mWcm^{-2}$) which is power of light absorbed by photoanode (maximum power of photoanode). Below formula shows such relationship for photon absorption (J_{abs}).

$$J_{abs}\left(\frac{mA}{cm^2}\right) = \int_{\lambda_1}^{\lambda_2} \frac{\lambda}{1240} P_{abs}(\lambda) d\lambda \quad \left(\frac{mW}{cm^2}\right) \quad (5)$$

J_{abs} is nearly avg. 5.08 (4.9~5.2) mA/cm^2 (avg. LHE = ~ 69.4%) for $BiVO_4$ films, while J_{max} = 7.5 mA/cm^2 when 100% of LHE till 2.4 eV (516 nm) threshold is assumed. Bulk and surface separation efficiencies are calculated by:

$$\eta_{bulk} = J^{SO3} / J_{abs} \quad (6)$$

$$\eta_{surf} = J^{H2O} / J^{SO3} \quad (7)$$

Supplementary References

1. Sayama, K. *et al.* Photoelectrochemical decomposition of water on nanocrystalline BiVO4 film electrodes under visible light. *Chem. Commun*, 2908-2909 (2003).
2. Sayama, K. *et al.* Effect of Carbonate Ions on the Photooxidation of Water over Porous BiVO4 Film Photoelectrode under Visible Light. *Chem. Lett.* **39**, 17-19 (2010).
3. Luo, W. *et al.* Solar hydrogen generation from seawater with a modified BiVO4 photoanode. *Energy Environ. Sci.* **4**, 4046-4051 (2011).
4. Abdi, F. F. *et al.* Efficient solar water splitting by enhanced charge separation in a bismuth vanadate-silicon tandem photoelectrode. *Nat. Commun.* **4**, 2195 (2013).
5. Han, L. *et al.* Efficient Water-Splitting Device Based on a Bismuth Vanadate Photoanode and Thin-Film Silicon Solar Cells. *ChemSusChem.* **7**, 2832-2838 (2014).
6. Kim, T. W. & Choi, K.-S. Nanoporous BiVO4 Photoanodes with Dual-Layer Oxygen Evolution Catalysts for Solar Water Splitting. *Science* **343**, 990-994 (2014).
7. Kim, J. H. *et al.* Wireless Solar Water Splitting Device with Robust Cobalt-Catalyzed, Dual-Doped BiVO4 Photoanode and Perovskite Solar Cell in Tandem: A Dual Absorber Artificial Leaf. *ACS Nano* **9**, 11820-11829 (2015).
8. Kim, T. W., Ping, Y., Galli, G. A. & Choi, K.-S. Simultaneous enhancements in photon absorption and charge transport of bismuth vanadate photoanodes for solar water splitting. *Nat. Commun.* **6**, 8769 (2015).
9. Beermann, N., Vayssieres, L., Lindquist, S. E. & Hagfeldt, A. Photoelectrochemical Studies of Oriented Nanorod Thin Films of Hematite. *J. Electrochem. Soc.***147**, 2456-2461 (2000).
10. Duret, A. & Grätzel, M. Visible Light-Induced Water Oxidation on Mesoscopic α-Fe2O3 Films Made by Ultrasonic Spray Pyrolysis. *J. Phys. Chem. B* **109**, 17184-17191 (2005).
11. Tilley, S. D., Cornuz, M., Sivula, K. & Grätzel, M. Light-Induced Water Splitting with Hematite: Improved Nanostructure and Iridium Oxide Catalysis. *Angew. Chem. Int.* **49**, 6405-6408, (2010).
12. Kim, J. Y. *et al.* Single-crystalline, wormlike hematite photoanodes for efficient solar water splitting. *Sci. Rep.* **3**, 2681 (2013).
13. Santato, C., Ulmann, M. & Augustynski, J. Photoelectrochemical Properties of Nanostructured Tungsten Trioxide Films. *J. Phys. Chem. B* **105**, 936-940 (2001).
14. Alexander, B. D., Kulesza, P. J., Rutkowska, I., Solarska, R. & Augustynski, J. Metal oxide photoanodes for solar hydrogen production. *J. Mater. Chem.* **18**, 2298-2303 (2008).
15. Li, W. *et al.* Visible light photoelectrochemical responsiveness of self-organized nanoporous WO3 films. *Electrochim. Acta.* **56**, 620-625 (2010).
16. Kim, J. K., Shin, K., Cho, S. M., Lee, T.-W. & Park, J. H. Synthesis of transparent mesoporous tungsten trioxide films with enhanced photoelectrochemical response: application to unassisted solar water splitting. *Energy Environ. Sci.* **4**, 1465-1470 (2011).
17. Su, J., Guo, L., Bao, N. & Grimes, C. A. Nanostructured WO3/BiVO4 Heterojunction Films for Efficient Photoelectrochemical Water Splitting. *Nano Lett.* **11**, 1928-1933 (2011).
18. Hong, S. J., Lee, S., Jang, J. S. & Lee, J. S. Heterojunction BiVO4/WO3 electrodes for enhanced photoactivity of water oxidation. *Energy Environ. Sci.* **4**, 1781-1787 (2011).
19. Saito, R., Miseki, Y. & Sayama, K. Highly efficient photoelectrochemical water splitting using a thin film photoanode of $BiVO_4/SnO_2/WO_3$ multi-composite in a carbonate electrolyte. *Chem. Commun* **48**, 3833-3835 (2012).
20. Shi, X. *et al.* Efficient photoelectrochemical hydrogen production from bismuth vanadate-decorated tungsten trioxide helix nanostructures. *Nat. Commun.* **5**, 4775 (2014).
21. Pihosh, Y. *et al.* Photocatalytic generation of hydrogen by core-shell $WO_3/BiVO_4$ nanorods with ultimate water splitting efficiency. *Sci. Rep.* **5**, 11141 (2015).
22. Miller, N. G. Y. C. J. K. A. D. E. L. Status of research on tungsten oxide-based photoelectrochemical devices at the University of Hawai'i. *SPIE* **7770** (2010).
23. Brillet, J. *et al.* Highly efficient water splitting by a dual-absorber tandem cell. *Nat Photon* **6**, 824-828 (2012).
24. Ding, C. *et al.* Solar-to-hydrogen efficiency exceeding 2.5% achieved for overall water splitting with an all earth-abundant dual-photoelectrode. *Phys. Chem. Chem. Phys.* **16**, 15608-15614 (2014).
25. Chen, Y.-S., Manser, J. S. & Kamat, P. V. All Solution-Processed Lead Halide Perovskite-$BiVO_4$ Tandem Assembly for Photolytic Solar Fuels Production. *J. Am. Chem. Soc.* **137**, 974-981 (2015).
26. Gurudayal *et al.* Perovskite–Hematite Tandem Cells for Efficient Overall Solar Driven Water Splitting. *Nano Lett.* **15**, 3833–3839 (2015).

27. Morales-Guio, C. G. *et al.* An Optically Transparent Iron Nickel Oxide Catalyst for Solar Water Splitting. *J. Am. Chem. Soc.* **137**, 9927-9936 (2015).
28. Fuku, K., Wang, N., Miseki, Y., Funaki, T. & Sayama, K. Photoelectrochemical Reaction for the Efficient Production of Hydrogen and High-Value-Added Oxidation Reagents. *ChemSusChem.* **8**, 1593-1600 (2015).
29. Luo, J. *et al.* Targeting Ideal Dual-Absorber Tandem Water Splitting Using Perovskite Photovoltaics and CuInxGa1-xSe2 Photocathodes. *Adv. Energy Mater.* **5**, 1-8 (2015).
30. Dias, P. *et al.* Transparent Cuprous Oxide Photocathode Enabling a Stacked Tandem Cell for Unbiased Water Splitting. *Adv. Energy Mater.* **5**, 1-9 (2015).
